# Luteolin Attenuates Cardiac Ischemia/Reperfusion Injury in Diabetic Rats by Modulating Nrf2 Antioxidative Function

**DOI:** 10.1155/2019/2719252

**Published:** 2019-04-08

**Authors:** Chi Xiao, Man-Li Xia, Jue Wang, Xin-Ru Zhou, Yang-Yun Lou, Li-Hui Tang, Feng-Jiang Zhang, Jin-Ting Yang, Ling-Bo Qian

**Affiliations:** ^1^School of Basic Medical Sciences & Forensic Medicine, Hangzhou Medical College, Hangzhou 310053, China; ^2^Institute of Physiological Function, Medical College of Jiaxing University, Jiaxing 314001, China; ^3^Department of Anesthesiology, the Second Affiliated Hospital, Zhejiang University School of Medicine, Hangzhou 310009, China

## Abstract

Luteolin has been reported to attenuate ischemia/reperfusion (I/R) injury in the diabetic heart through endothelial nitric oxide synthase- (eNOS-) related antioxidative response. Though the nuclear factor erythroid 2-related factor 2 (Nrf2) is regarded as a key endogenous factor to reduce diabetic oxidative stress, whether luteolin reduces cardiac I/R injury in the diabetic heart via enhancing Nrf2 function needs to be clarified. We hypothesized that pretreatment with luteolin could alleviate cardiac I/R injury in the diabetic heart by affecting the eNOS/Nrf2 signaling pathway. The diabetic rat was produced by a single injection of streptozotocin (65 mg/kg, i.p.) for 6 weeks, and then, luteolin (100 mg/kg/day, i.g.), eNOS inhibitor L-NAME, or Nrf2 inhibitor brusatol was administered for the succedent 2 weeks. After that, the isolated rat heart was exposed to 30 min of global ischemia and 120 min of reperfusion to establish I/R injury. Luteolin markedly ameliorated cardiac function and myocardial viability; upregulated expressions of heme oxygenase-1, superoxide dismutase, glutathione peroxidase, and catalase; and reduced myocardial lactate dehydrogenase release, malondialdehyde, and 8-hydroxydeoxyguanosine in the diabetic I/R heart. All these ameliorating effects of luteolin were significantly reversed by L-NAME or brusatol. Luteolin also markedly reduced *S*-nitrosylation of Kelch-like ECH-associated protein 1 (Keap1) and upregulated Nrf2 and its transcriptional activity. This effect of luteolin on Keap1/Nrf2 signaling was attenuated by L-NAME. These data reveal that luteolin protects the diabetic heart against I/R injury by enhancing eNOS-mediated *S*-nitrosylation of Keap1, with subsequent upregulation of Nrf2 and the Nrf2-related antioxidative signaling pathway.

## 1. Introduction

In 2017, about 425 million adults aged 20-79 years had diabetes, and these numbers were expected to increase to 629 million by 2045 [1]. The risk of cardiovascular disease, including myocardial infarction, heart failure, and stroke, and the overall mortality are twofold to fourfold greater in diabetic patients than in nondiabetic ones according to the Framingham Heart Study [2, 3]. Moreover, the clinical prognosis after myocardial infarction in diabetic patients is usually poorer than that in nondiabetic subjects [4]. Ischemic conditioning and agents that protect the heart against ischemia/reperfusion (I/R) injury in nondiabetic subjects are mostly resisted in diabetic animals and patients [5]. These data suggest that the loss of endogenous protective mechanisms either by hyperglycemia or by diabetes itself may result in the poor prognosis in diabetic subjects after myocardial I/R events and vigorous therapeutic strategies targeting I/R injury are required to benefit this population.

An excess of reactive oxygen species (ROS) induced by hyperglycemia contributes to the enhanced basal oxidative stress and is likely to aggravate myocardial I/R injury in diabetic patients [4, 6]. Defective intracellular antioxidase production and activity, such as superoxide dismutase (SOD) and glutathione peroxidase (GPx), also contributes to increased oxidative stress in diabetes [7]. Such a higher basal level of oxidative stress causes protein carboxylation, lipid peroxidation, and DNA damage, ultimately leading to cell death and contractile dysfunction in the diabetic heart [8]. Thus, improving antioxidase expression and suppressing oxidative stress are appropriate approaches in treating diabetic cardiovascular disease. The nuclear factor erythroid 2-related factor 2 (Nrf2), widely expressed in the liver, lung, heart, and brain, is regarded as the most important endogenous factor associated with the cellular response to oxidative stress [9]. Nrf2 translocates into the nucleus and binds with an antioxidant response element (ARE) to upregulate a battery of antioxidative gene expressions, including heme oxygenase-1 (HO-1), SOD, catalase, and GPx, in response to antioxidants and oxidative stress and then reduces the cardiac injury [10, 11]. Several studies have reported that Nrf2 antioxidative dysfunction occurs in diabetes and activation of Nrf2 clearly upregulates HO-1, SOD, and GPx expressions to reduce diabetic cardiomyopathy and myocardial I/R injury [12–15]. It is noteworthy that the endothelial nitric oxide synthase- (eNOS-) NO pathway is shown to be associated with the Nrf2-related decrease of cardiac oxidative stress in diabetic rats and eNOS activity is essential for the Nrf2-dependent cardioprotection against hypoxia/reoxygenation injury [13, 16]. Furthermore, *S*-nitrosylation of Kelch-like ECH-associated protein 1 (Keap1), the inhibitor of Nrf2, by eNOS allows Nrf2 to translocate into the nucleus to induce transcription of a variety of antioxidative genes, with a resultant attenuation of I/R oxidative stress in the hippocampus [17]. These results indicate that eNOS activation might protect the diabetic heart against I/R injury through the Nrf2 antioxidative signaling pathway.

As a common dietary source, the polyphenolic compound luteolin is widely contained in vegetables, such as celery, broccoli, green pepper, and other dietary plants [18–20]. Our previous study revealed that pretreatment with luteolin can diminish myocardial I/R injury in the early diabetic (5 weeks) rat by activating eNOS and inhibiting mitochondrial oxidative stress [21]. However, the cardioprotection and mechanism that mediate the antioxidative effect of luteolin on midterm diabetic (8 weeks) hearts exposed to I/R insult remain unclear. Other reports showed that luteolin can protect the heart against isoproterenol-induced damage through the activation of the Nrf2 antioxidative pathway [22]. This Nrf2-related antioxidative effect of luteolin has also been demonstrated in the brain and liver [23, 24]. Thus, we may speculate that the antioxidative effect of luteolin on diabetic I/R hearts may be associated with the activation of eNOS and the subsequent Nrf2 function.

Here, we hypothesized that pretreatment with luteolin can attenuate myocardial I/R injury in the diabetic rat by eNOS activation and the *S*-nitrosylation of Keap1, with subsequent upregulation of the Nrf2 antioxidative pathway and a resultant alleviation of oxidative stress.

## 2. Materials and Methods

### 2.1. Reagents

Luteolin, streptozotocin (STZ), N*^ω^*-nitro-L-arginine methyl ester hydrochloride (L-NAME), 3-(4,5-dimethyl-2-thiazolyl)-2,5-diphenyl-2H-tetrazolium bromide (MTT), and dimethyl sulfoxide (DMSO) were purchased from Sigma-Aldrich (Saint Louis, MO, USA). The nuclear extraction kit and antibodies to Nrf2 and Keap1 were from Abcam (Cambridge, MA, USA). The antibody to histone H3 was from Cell Signaling Technology (Beverly, MA, USA). Primers of HO-1, SOD, GPx, catalase, and GAPDH for PCR were purchased from Thermo Fisher (Grand Island, NY, USA). The *S*-nitrosylated protein detection kit (Biotin Switch) was from Cayman Chemical (Ann Arbor, MI, USA). DNA binding ELISA for activated Nrf2 (TransAM® Nrf2) was purchased from Active Motif (Carlsbad, CA, USA). ELISA kits for 8-hydroxydeoxyguanosine (8-OHdG) were from USCN Life Science (Wuhan, China). The kits for measurement of lactate dehydrogenase (LDH) and malondialdehyde (MDA) were from the Nanjing Jiancheng Bioengineering Institute (Nanjing, China). Luteolin was dissolved in 0.5% (*w*/*v*) sodium carboxymethyl cellulose (CMC-Na). All other reagents were of analytical purity.

### 2.2. Diabetic Rat Model

Male Sprague-Dawley rats (220 ± 10 g), purchased from the Shanghai Laboratory Animal Center, were housed under a 12 h light/12 h dark and 22°C environment and fed on water and standard pellet chow ad libitum. All animal procedures were carried out according to the Guide for the Care and Use of Laboratory Animals published by the US NIH (the 8th edition, NRC 2011) and were approved by the Ethics Committee for the Use of Experimental Animals in Hangzhou Medical College. Diabetes was induced by a single dose of STZ (65 mg/kg, i.p.) according to our previous report [21]. Fasting blood glucose was measured 72 h after STZ administration using a OneTouch glucometer. Rats with blood glucose more than16.7 mM were considered to be diabetic.

### 2.3. Experimental Protocols

Rats were randomly divided into five groups (7 per group): (1) nondiabetic controls (ND), (2) STZ diabetic rats (D), (3) STZ diabetic rats treated with luteolin (100 mg/kg/d, i.g.) [21, 25] (D+Lut), (4) STZ diabetic rats treated with luteolin and the NOS inhibitor L-NAME (25 mg/kg/d, i.g.) [21, 26] (D+L-NAME+Lut), and (5) STZ diabetic rats treated with luteolin and the Nrf2 inhibitor brusatol (0.4 mg/kg/2d, i.p.) [27] (D+Brusatol+Lut). L-NAME and luteolin were administered intragastrically for 2 consecutive weeks, beginning 6 weeks after STZ injection.

### 2.4. Preparation of the I/R Model in the Isolated Rat Heart

As described in our previous work [21, 28], at the end of 8 weeks, the rat was anesthetized and the heart was rapidly excised to be retrogradely perfused on a Langendorff apparatus with modified Krebs-Henseleit buffer (pH 7.4, 37°C) containing the following (in mM): NaCl 118.0, NaHCO_3_ 25.0, KCl 4.7, CaCl_2_ 1.25, KH_2_PO_4_ 1.2, MgSO_4_ 1.2, and glucose 11.0, equilibrated with 95% O_2_ + 5% CO_2_. After equilibration for 20 min, the heart was subjected to global ischemia for 30 min and reperfusion for 120 min. Left ventricular developed pressure (LVDP), LV end diastolic pressure (LVEDP), and heart rate (HR) were recorded throughout the experimental period. Rate pressure product (RPP) means LVDP × HR. After completing reperfusion, the heart was cut transversely into 3 parts for determining tissue viability, MDA level, and molecular expressions.

### 2.5. Determination of Cardiac Tissue Viability, LDH Release, MDA, and 8-OHdG

After reperfusion for 120 min, the heart was cut into 2 mm thick slices and incubated with MTT (3 mM) at 37°C for 30 min to form formazan. Viability was determined by the level of formazan using a spectrophotometer at 550 nm. Based on previous studies from our own and other labs [21, 28–31], cardiac LDH release at 5 min of reperfusion in the coronary effluent was determined by spectrophotometry following the commercial kit manual. After reperfusion for 120 min, the myocardial MDA level was determined by spectrophotometry according to the commercial kit manual [21, 28]. The levels of 8-OHdG were measured with ELISA kits according to the manufacturer's instructions [32, 33].

### 2.6. Western Blotting

At the end of reperfusion, the expression of myocardial Keap1 was determined by Western blotting as we previously reported [21, 28, 34]. Nuclear protein was isolated from the heart tissue using a nuclear extraction kit following the manufacturer's instructions. Nuclear Nrf2 was detected by Western blotting [28, 35].

### 2.7. Determination of *S*-Nitrosylation of Keap1

S-Nitrosylation of Keap1 was determined using the *S*-nitrosylated protein detection kit based on the biotin switch method [36]. Briefly, after reperfusion, the heart tissue was lysed to extract proteins. The heart protein was immunoprecipitated with anti-Keap1 antibody. After immunoprecipitation, pellets were handled using the *S*-nitrosylated protein detection kit (Biotin Switch) following the manufacturer's instruction to detect the *S*-nitrosylation level [17, 37].

### 2.8. Determination of Nrf2-DNA Binding Activity

The DNA binding activity of Nrf2 was measured using the TransAM® Nrf2 kit following the manufacturer's protocol and expressed as fold changes in absorbance compared with that in the ND group [17].

### 2.9. Reverse Transcription and Quantitative PCR

Total RNA purification, reverse transcription, and quantitative PCR were performed as described before [28, 34] using the following primers: HO-1, SOD, GPx, catalase, and GAPDH.

### 2.10. Statistical Analysis

Data are presented as mean ± SD. Differences were made using one-way ANOVA followed by the Newman-Keuls test (GraphPad Prism 5.0, San Diego, CA, USA). *P* < 0.05 was considered a significant difference.

## 3. Results

### 3.1. Effect of Luteolin on Blood Glucose

**Figure 1:**
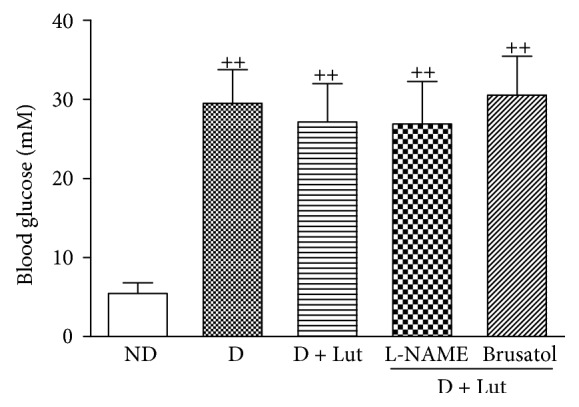
Effect of luteolin on blood glucose in diabetic rats. ND: nondiabetic rats; D: diabetic rats; D+Lut: diabetic rats treated with luteolin (100 mg/kg/d, i.g.); D+L-NAME+Lut: diabetic rats treated with the NOS inhibitor L-NAME (25 mg/kg/d, i.g.) and luteolin; D+Brusatol+Lut: STZ diabetic rats treated with brusatol (0.4 mg/kg/2d, i.p.) and luteolin. Six weeks after STZ injection, L-NAME, brusatol, and luteolin were administered for two weeks. All data are expressed as mean ± SD; *n* = 10 rats per group; ^++^*P* < 0.01 versus ND.

After 8 weeks, the blood glucose in the D group was still markedly increased compared with that in the ND group (*P* < 0.01). Luteolin, L-NAME, and brusatol did not significantly affect the blood glucose in any of the diabetic groups (Figure 1).

### 3.2. Effect of Luteolin on Left Ventricular Function

As shown in Table 1, the baseline RPP was significantly decreased in the D group compared with the ND group (*P* < 0.05), which was markedly reversed by treatment with luteolin (*P* < 0.05 versus the D group). This effect of luteolin was attenuated by treatment with L-NAME and brusatol (*P* < 0.05 versus the D+Lut group). After reperfusion, the RPP in the D group was markedly lower than that in ND rats (*P* < 0.01) and this was reversed by pretreatment with luteolin (*P* < 0.01 versus the D group). However, both L-NAME and brusatol attenuated the ameliorating effect of luteolin on RPP in the diabetic I/R heart (*P* < 0.01 versus the D+Lut group). At the end of reperfusion, LVEDP was significantly increased in the D group compared with the ND group (*P* < 0.01) and this was significantly attenuated by pretreatment with luteolin (*P* < 0.01 versus the D group). Similarly, the ameliorating effect of luteolin on LVEDP was abolished by L-NAME and brusatol (*P* < 0.01 versus the D+Lut group).

### 3.3. Effect of Luteolin on Cardiac Tissue Viability and LDH Release

**Figure 2:**
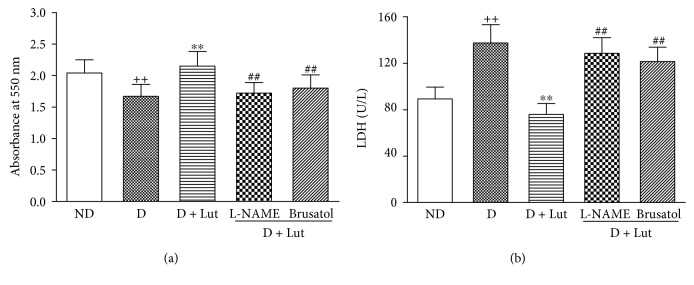
Effect of luteolin on myocardial viability as indicated by formazan content at the end of reperfusion (a) and LDH release in the coronary effluent during reperfusion (b) in diabetic rats. The hearts isolated from the five groups were exposed to 30 minutes of global ischemia and 120 minutes of reperfusion. All data are expressed as mean ± SD; *n* = 10 rats per group; ^++^*P* < 0.01 versus ND; ^∗∗^*P* < 0.01 versus D; ^##^*P* < 0.01 versus D+Lut.

The decrease of cardiac tissue viability and the increase of myocardial LDH release in the D group induced by I/R were both significantly reversed by pretreatment with luteolin (*P* < 0.01; Figure 2). These ameliorating effects of luteolin in the diabetic I/R rat heart were abolished by L-NAME and brusatol (*P* < 0.01).

### 3.4. Effect of Luteolin on Myocardial MDA, 8-OHdG, and Nrf2 Function

**Figure 3:**
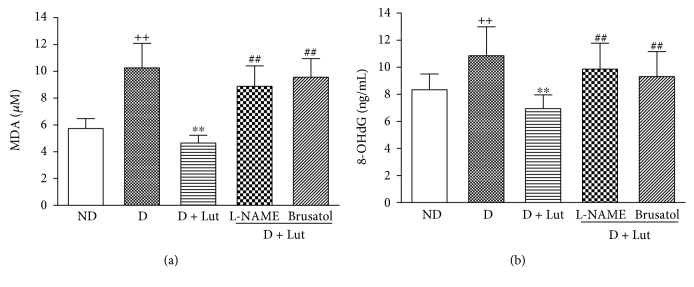
Effect of luteolin on myocardial MDA (a) and 8-OHdG (b) in rat hearts from all five groups exposed to 30 minutes of global ischemia and 120 minutes of reperfusion. All data are expressed as mean ± SD; *n* = 10 rats per group; ^++^*P* < 0.01 versus ND; ^∗∗^*P* < 0.01 versus D; ^##^*P* < 0.01 versus D+Lut.

**Figure 4:**
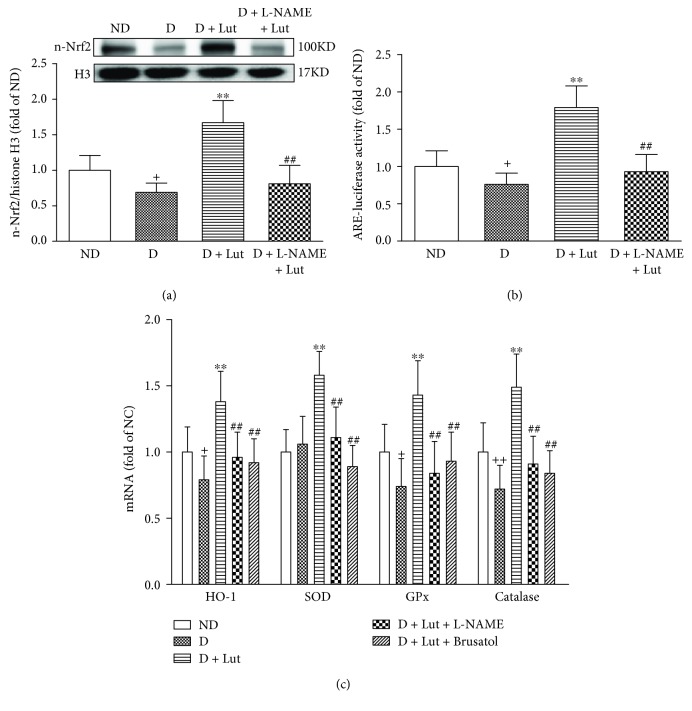
Effect of luteolin on the nuclear translocation of Nrf2 (a), DNA binding activity of Nrf2 (b), and Nrf2-modulated antioxidative mRNA expressions (c) in rat hearts exposed to 30 minutes of global ischemia and 120 minutes of reperfusion. All data are expressed as mean ± SD; *n* = 10 rats per group; ^+^*P* < 0.05 versus ND; ^∗∗^*P* < 0.01 versus D; ^##^*P* < 0.01 versus D+Lut.

The increase of myocardial MDA in the D group at the end of reperfusion was significantly reversed by pretreatment with luteolin (*P* < 0.01; Figure 3(a)). This effect of luteolin on MDA was abolished by L-NAME and brusatol (*P* < 0.01). Similar results were found in myocardial 8-OHdG (Figure 3(b)). The decrease of nuclear Nrf2 in the diabetic heart subjected to I/R was markedly reversed by pretreatment with luteolin (*P* < 0.01; Figure 4(a)). The DNA binding activity of Nrf2 was significantly increased by luteolin compared with that in the D group (*P* < 0.01; Figure 4(b)). Nrf2 downstream antioxidative gene HO-1, SOD, GPx, and catalase mRNA expressions were also markedly increased by luteolin compared with that in the D group (*P* < 0.01; Figure 4(c)). All these improvements of luteolin were inhibited by L-NAME and brusatol (*P* < 0.01; Figure 4).

### 3.5. Effect of Luteolin on Myocardial *S*-Nitrosylation of Keap1

**Figure 5:**
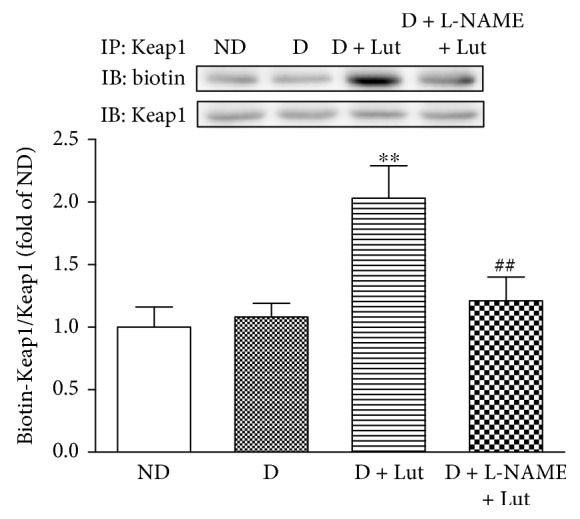
Effect of luteolin on *S*-nitrosylation of Keap1 in rat hearts exposed to 30 minutes of global ischemia and 120 minutes of reperfusion. All data are expressed as mean ± SD; *n* = 10 rats per group; ^∗∗^*P* < 0.01 versus D; ^##^*P* < 0.01 versus D+Lut.

The decrease of myocardial *S*-nitrosylation of Keap1 in the D group at the end of reperfusion was significantly reversed by pretreatment with luteolin (*P* < 0.01). This effect of luteolin on the *S*-nitrosylation of Keap1 was abolished by L-NAME (*P* < 0.01; Figure 5).

## 4. Discussion

**Figure 6:**
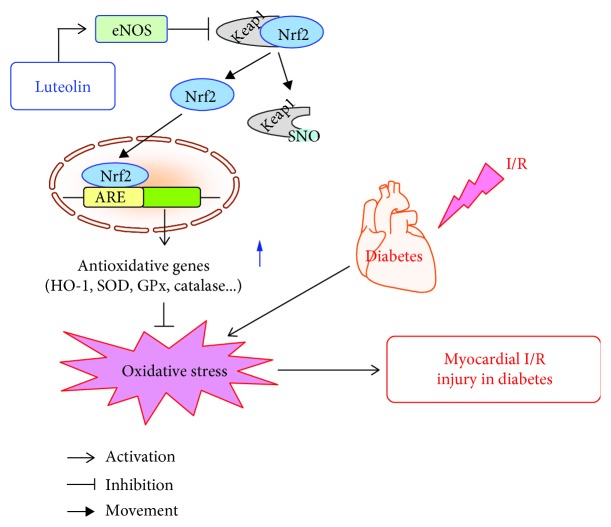
Schematic diagram for the cardioprotection of luteolin against I/R injury in the diabetic heart. Luteolin initiates nuclear translocation and activation of Nrf2 by activating eNOS and the subsequent *S*-nitrosylation and inhibition of Keap1, which upregulates the antioxidative gene (HO-1, SOD, GPx, and catalase) expression and inhibits oxidative stress, leading to the decrease of diabetic myocardial I/R injury.

In the current work, we found that (1) treatment with luteolin attenuated myocardial injury and improved cardiac function in the I/R heart from the STZ-induced diabetic rat at the 8-week point, (2) activation of the Nrf2 antioxidative function was essential for this action of luteolin as proved by the evidence that brusatol, an inhibitor of Nrf2, reduced the cardioprotective effect of luteolin, and (3) luteolin protected the diabetic heart against I/R injury through *S*-nitrosylation of Keap1 and then activation of Nrf2 as demonstrated by the evidence that the eNOS inhibitor L-NAME decreased the *S*-nitrosylation of Keap1, Nrf2 function, and cardioprotection induced by luteolin (Figure 6). Consistent with our previous studies, the 2-week treatment with luteolin did not significantly affect blood glucose in the diabetic rat [21]. These results suggest that luteolin protects the diabetic heart against I/R injury mainly through enhancing Nrf2 function and reducing oxidative stress.

There are some mechanisms involved in the pathogenetic process of diabetic cardiac dysfunction, including high oxidative stress [38]. The excessive ROS production in diabetes can oxidize cellular proteins, membrane lipids, and nucleic acids to aggravate cardiac injury following myocardial I/R [39]. The increase in ROS production is associated with the decrease in endogenous antioxidative defenses in diabetic subjects, and supplementary antioxidants, such as vitamins (C, E), glutathione, *α*-lipoic acid, and trace elements, have been demonstrated to improve cardiovascular function in diabetic animal models [7]. In the current study, we found that systolic and diastolic functions in the diabetic heart were significantly impaired at the end of reperfusion accompanying the decrease in cell viability and the increase in LDH release. Pretreatment with luteolin not only improved the cardiac function and myocardial viability but also reduced the myocardial MDA and 8-OHdG in the diabetic rat heart exposed to I/R procedure. MDA and 8-OHdG are the best known biomarkers of lipid and DNA peroxidation, respectively, indicating lipid and DNA damages caused by excessive ROS [32, 33]. Further experiments showed that luteolin upregulated antioxidative enzymes HO-1, SOD, GPx, and catalase in the diabetic I/R heart. Based on our previous study that luteolin enhances SOD activity and inhibits mitochondrial permeability transition pore opening in the I/R-treated diabetic heart, these results further suggest that luteolin protects the diabetic rat heart against I/R injury through scavenging ROS and enhancing the cellular antioxidative system.

The upregulated expression of antioxidative enzymes in an Nrf2-dependent manner has been clarified. Nrf2 transcriptionally induces several antioxidative genes including HO-1, SOD, and GPx and catalase to activate cytoprotective pathways against inflammation and oxidative injury [40]. Evidence from a great many animal and cell models points out a key role of Nrf2 in attenuation of diabetes and diabetic complications [41–43]. Previous studies revealed that activating the Nrf2 signaling pathway mediates the cardioprotection of luteolin against oxidative damage [22, 44]. This Nrf2-involved protective effect of luteolin was also demonstrated in diabetic nervous systems and hypercholesterolemic I/R hearts [28, 45]. Therefore, it is of particular interest to explore whether luteolin can attenuate I/R injury in the diabetic heart through activation of the Nrf2-related antioxidative pathway. Here, we found that luteolin significantly increased the translocation of Nrf2 into the nucleus correlated with a significant enhancement in ARE binding activity of Nrf2 and Nrf2-modulated antioxidative gene expressions in the diabetic heart exposed to I/R insult. Furthermore, the Nrf2 inhibitor brusatol reversed the cardioprotection of luteolin in diabetic I/R hearts. These results indicate that luteolin alleviates the cardiac I/R injury in the diabetic rat, at least, partly through the activation of the Nrf2 antioxidative signaling pathway. However, the underlying mechanism that mediates the activation of Nrf2 by luteolin in the diabetic I/R rat heart needs to be clarified.

Nrf2 is normally bound to Keap1 to form a cytosolic complex, resulting in its ubiquitination and degradation [46]. It has been demonstrated that activation of eNOS and neuronal NOS can produce NO to trigger Nrf2-dependent antioxidative response through *S*-nitrosylation of Keap1 cysteine residues, which protects the neurocyte against oxidative stress injury [17, 47]. Activation of eNOS has already been shown to diminish cardiac oxidative stress injury in several animal I/R models, and this protection can be abolished by the eNOS inhibitor L-NAME though the exact target for eNOS remains elusive [48–50]. Intriguingly, we have previously found that pretreatment with luteolin has beneficial effects on the early diabetic rat heart subjected to I/R procedure through activation of the eNOS pathway [21]. In the current work, the cardioprotection and Nrf2-related antioxidative enzyme expression conferred by luteolin in the midterm diabetic heart were blocked by L-NAME. Furthermore, luteolin-induced nuclear localization of Nrf2 and *S*-nitrosylation of Keap1 in the diabetic I/R heart were significantly hampered by L-NAME. These results indicate that activation of eNOS and the subsequent *S*-nitrosylation of Keap1 are involved in the Nrf2-modulated antioxidative function aroused by luteolin in the diabetic heart. Although we reported in the previous study that luteolin can reduce I/R injury in the early diabetic (5 weeks) rat heart via enhancing eNOS activity and reducing oxidative stress [21], the effect of luteolin on the midterm or late diabetic heart subjected to I/R insult remains unclear. The mechanism by which luteolin reduces oxidative stress in the diabetic I/R heart through eNOS signaling also needs to be clarified. The newly found result in the current work is not only that luteolin protects the early diabetic (5 weeks) heart but also that the midterm diabetic (8 weeks) heart underwent I/R procedure. Furthermore, we firstly found that this protective effect of luteolin on the midterm diabetic rat heart associates with eNOS-Keap1-Nrf2-related antioxidative signaling. However, the detailed molecular mechanism that activates eNOS by luteolin remains unclear. The extensive ROS produced by diabetes might oxidize and inactivate eNOS, finally leading to myocardial dysfunction and even cell death [49, 51]. It is reasonable to deduce that eNOS activity is downregulated by the diabetes-induced oxidative stress, which is reversed by the antioxidative action of luteolin during reperfusion to reduce further the cardiac I/R oxidative injury. Although it is still unclear how luteolin induces eNOS activation in the diabetic I/R heart, the possibility that luteolin may interact with cellular signaling transduction pathways, such as the cAMP/PKA-mediated mechanism [52, 53], to modulate eNOS activity cannot be neglected.

## 5. Conclusions

In conclusion, the current work suggests that luteolin can reduce oxidative stress to profoundly protect the diabetic rat heart against I/R injury through enhancing eNOS-mediated *S*-nitrosylation of Keap1, with subsequent upregulation of nuclear Nrf2 and the Nrf2-related antioxidative signaling pathway.

## Figures and Tables

**Table 1:** Effect of luteolin on left ventricular hemodynamic parameters in diabetic rat hearts exposed to ischemia/reperfusion.

Variable	Baseline	Reperfusion (min)		
		30	60	120
*RPP (% of baseline)*				
ND	100 (25273.5 ± 2724.7 mmHg beats/min)	51.4 ± 6.9	42.3 ± 5.6	33.9 ± 4.8
D	100 (21144.6 ± 2424.1 mmHg beats/min)^+^	52.7 ± 7.5	41.2 ± 6.2	23.6 ± 4.2^++^
D+Lut	100 (24603.7 ± 2304.3 mmHg beats/min)^∗^	53.4 ± 7.1	54.6±7.2^∗∗^	40.7±6.5^∗∗^
D+L-NAME+Lut	100 (21935.7 ± 2064.5 mmHg beats/min)^#^	53.1 ± 6.7	43.4 ± 7.5^##^	28.5 ± 4.9^##^
D+Brusatol+Lut	100 (20985.4 ± 2103.6 mmHg beats/min)^#^	50.4 ± 7.6	40.5 ± 6.9^##^	26.3 ± 3.7^##^
*LVEDP (mmHg)*				
ND	6.2 ± 0.5	27.1 ± 3.2	29.3 ± 3.5	28.3 ± 3.3
D	6.3 ± 0.6	29.4 ± 3.8	30.5 ± 3.2	34.5 ± 3.9^++^
D+Lut	6.4 ± 0.7	25.1 ± 3.6^∗^	27.2 ± 3.1^∗^	26.1±3.5^∗∗^
D+L-NAME+Lut	6.3 ± 0.4	30.1 ± 3.6^#^	32.6 ± 3.7^#^	33.4 ± 3.8^##^
D+Brusatol+Lut	6.5 ± 0.8	29.7 ± 3.2^#^	30.3 ± 3.3^#^	32.9 ± 3.4^##^

Data are mean ± SD, *n* = 10 rats/group. ND: nondiabetic rats; D: STZ diabetic rats; D+Lut: STZ diabetic rats treated with luteolin (100 mg/kg/d); D+L-NAME+Lut: diabetic rats treated with the NOS inhibitor L-NAME (25 mg/kg/d) and luteolin; D+Brusatol+Lut: diabetic rats treated with the Nrf2 inhibitor brusatol (0.4 mg/kg/2d) and luteolin. Six weeks after STZ injection, L-NAME and luteolin were administered intragastrically for 2 weeks. Hearts were isolated and subjected to global ischemia for 30 min and reperfusion for 120 min. ^+^*P* < 0.05 and ^++^*P* < 0.01 versus ND; ^∗^*P* < 0.05 and ^∗∗^*P* < 0.01 versus D; ^##^*P* < 0.01 versus D+Lut.

## Data Availability

The data used to support the findings of this study are included within the article.
